# Comparative Pharmacokinetic Analysis of Aflibercept and Brolucizumab in Human Aqueous Humor Using Nano-Surface and Molecular-Orientation Limited Proteolysis

**DOI:** 10.3390/ijms26020556

**Published:** 2025-01-10

**Authors:** Kosuke Nagaoka, Natsuka Kimura, Satoru Inoda, Takuya Takayama, Yusuke Arai, Yasuo Yanagi, Takashi Shimada, Ryozo Nagai, Hidenori Takahashi, Kenichi Aizawa

**Affiliations:** 1 Department of Ophthalmology, Jichi Medical University, Shimotsuke-shi 329-0498, Tochigi, Japan; 2 Division of Clinical Pharmacology, Department of Pharmacology, Jichi Medical University, Shimotsuke-shi 329-0498, Tochigi, Japan; 3 Department of Ophthalmology and Micro-Technology, Yokohama City University, Yokohama-shi 232-0023, Kanagawa, Japan; 4 Singapore National Eye Centre, Singapore Eye Research Institute, Singapore 168751, Singapore; 5 Technology Research Laboratory, Shimadzu Corporation, Kyoto-shi 604-8511, Kyoto, Japan; 6 Jichi Medical University, Shimotsuke-shi 329-0498, Tochigi, Japan; 7 Clinical Pharmacology Center, Jichi Medical University Hospital, Shimotsuke-shi 329-0498, Tochigi, Japan; 8 Division of Translational Research, Clinical Research Center, Jichi Medical University Hospital, Shimotsuke-shi 329-0498, Tochigi, Japan

**Keywords:** aflibercept, brolucizumab, human, half-life, intravitreal injection, vitreous humor, LC-MS/MS, nSMOL

## Abstract

Aflibercept and brolucizumab, two anti-VEGF agents used as intravitreal injections in ophthalmology, differ significantly in molecular weight (aflibercept—115 kDa and brolucizumab—26 kDa). Using aqueous humor samples collected after drug administration, we measured and performed a comparative analysis of pharmacokinetics and half-lives of these drugs in the human eye. Since the quantification of monoclonal antibodies (mAbs) using antigen–antibody reactions, such as ELISA, is influenced by endogenous ligands or anti-drug antibodies, we employed nano-surface and molecular-orientation limited proteolysis (nSMOL), combined with liquid chromatography–tandem mass spectrometry (LC-MS/MS), for accurate measurements. Aqueous humor samples were collected from 59 eyes of 59 patients treated with aflibercept and 52 eyes of 52 patients treated with brolucizumab. Samples were obtained with a median post-injection period of 30 (range, 2–49) days for aflibercept and 28 (range, 4–60) days for brolucizumab. A population pharmacokinetic (PPK) analysis revealed that the half-life of aflibercept in human aqueous humor was significantly shorter than that of brolucizumab, 2.88 days versus 9.00 days, respectively (*p* = 1.16 × 10^−7^). Using the same mass spectrometry conditions, we calculated the half-lives of the two drugs. These results may be useful for optimizing the efficacy of these drugs in clinical practice.

## 1. Introduction

Aflibercept (Regeneron Pharmaceuticals, Inc., Tarrytown, NY, USA) and brolucizumab (Novartis Biopharma, Basel, Switzerland) are anti-vascular, endothelial growth factor (VEGF) drugs [1,2] that are used to treat various forms of neovascularization and vascular leakage in the eye [3,4,5]. Aflibercept finds applications in treating conditions such as neovascular, age-related macular degeneration (nAMD) [6], diabetic macular edema (DME) [7,8], myopic choroidal neovascularization (mCNV), macular edema associated with retinal vein occlusion (RVO), neovascular glaucoma (NVG), and retinopathy of prematurity (ROP). Molecularly, aflibercept consists of the extracellular binding domains of VEGF receptors 1 and 2 fused to an immunoglobulin Fc domain [9,10], and its molecular weight is 115 kDa. By binding to VEGF-A, VEGF-B, and PlGF, aflibercept inhibits the activation of VEGFR-1 and VEGFR-2, thereby suppressing inflammation, angiogenesis, and increased vascular permeability. Aflibercept reportedly maintains significant intravitreal VEGF-binding activity for 10–12 weeks after a single injection [11]. Recently, high-dose aflibercept with a higher concentration was approved for nAMD and DME by the U.S. Food and Drug Administration (FDA) [12].

Brolucizumab is another anti-VEGF drug with a reportedly longer-lasting effect compared to the more commonly used aflibercept [13]. Brolucizumab is approved only for nAMD [14] and DME [15]. It is a humanized, single-chain antibody fragment scFv without an immunoglobulin Fc domain, consisting of complementarity-determining regions (H and L chains) that bind to VEGF-A. The molecular weight of brolucizumab is 26 kDa, but its molar concentration is approximately 23 times that of aflibercept. By binding to VEGF-A, brolucizumab inhibits the activation of VEGFR-1 and VEGFR-2. Brolucizumab reportedly achieves a higher concentration in the RPE/choroid and exerts strong anti-VEGF activity that also allows for a treatment interval of 12 weeks [14]. Clinical trials and real-world reports have shown favorable anatomical outcomes in the treatment of nAMD. Specifically, a more rapid reduction in fluid pressure is observed compared to aflibercept; however, intraocular inflammation occurs at a relatively high rate of 4.6% [16]. Since the molecular weight of brolucizumab (26 kDa) is less than those of other anti-VEGF drugs, including aflibercept (115 kDa), its half-life is expected to be shorter [17]. To achieve higher efficacy, it is given at a higher dose; however, intravitreally, it may form aggregates due to the highly concentrated formulation. Non-native species in these aggregates are thought to contribute to the higher rate of inflammation [18].

From the standpoint of drug efficacy and side effects, the pharmacokinetics of these two drugs are important. In the past, the measurement of anti-VEGF agents has relied primarily on antigen–antibody reactions, such as ELISA. We have previously shown that for VEGF, various anti-VEGF agents interfere with the measurement of VEGF concentrations determined by ELISA [19]. In brief, while antigen–antibody reactions can be extremely sensitive using standard concentrations, complications may arise, especially when there is a protein that competitively binds close to the epitope of the monoclonal antibody. In such cases, binding disruptions may result in spuriously low concentrations, especially at lower concentrations. As such, using ELISA may incorrectly estimate the half-lives of anti-VEGF drugs. To circumvent artifacts introduced by this inhibition, we developed alternative measurement techniques, i.e., mass spectrometry, for the measurement of aflibercept [20,21].

In this study, we developed a highly sensitive MS method for brolucizumab under conditions consistent with those previously established for aflibercept, and we measured the aqueous humor concentration of brolucizumab. Using a previously reported method, we also measured the aqueous humor concentration of aflibercept and calculated and compared the intraocular half-lives of the two drugs. By employing the same measurement method for both drugs, this approach provides a robust basis for comparisons. Furthermore, differences in half-life may explain the observed differences in efficacy and the duration of effect between the two drugs in clinical practice.

## 2. Results

### 2.1. Patient Characteristics

One hundred and eleven eyes of 111 patients undergoing their first intravitreal injection with aflibercept or brolucizumab from March 2013 to August 2021 were studied. A total of 59 eyes from 59 patients were included in the aflibercept group, while the brolucizumab group comprised 52 eyes from 52 patients. The mean age (±standard deviation) was 70.8 ± 10.2 years in the aflibercept group and 74.6 ± 7.2 years in the brolucizumab group, showing a statistically significant difference (*p* = 0.03). The prevalence of posterior vitreous detachment (PVD) was observed in 21 eyes (36%) in the aflibercept group and 32 eyes (62%) in the brolucizumab group, showing a significant difference (*p* = 0.006). No significant differences were observed in terms of gender or axial length between the two groups (Table 1).

### 2.2. Summary of Measurements

From the initial injection, the median intervals for sample collection were 30 (IQR: 26–35, range: 2–49) days for aflibercept and 28 (IQR: 28, range: 4–60) days for brolucizumab. The median concentrations in aqueous humor were 5.3 (IQR: 1.05–12.6) µg/mL for aflibercept and 31.4 (IQR: 15.6–74.4) µg/mL for brolucizumab. There were 13 samples for aflibercept and 25 samples for brolucizumab that were below the limit of quantitation. For these samples, we calculated the values using half of the limit of detection (LOD), which was 1.05 μg/mL for aflibercept and 15.625 μg/mL for brolucizumab.

For curve fitting to calculate the half-life, the corrected Akaike information criterion (AICc) for the one-compartment model was 281 for aflibercept and 525 for brolucizumab, and the Bayesian information criterion (BIC) was 288 for aflibercept and 531 for brolucizumab. In contrast, the AICc for the two-compartment model was 283 for aflibercept and 527 for brolucizumab. The BIC was 291 for aflibercept and 535 for brolucizumab. Therefore, we adopted the one-compartment model, as it had smaller values.

**Figure 1:**
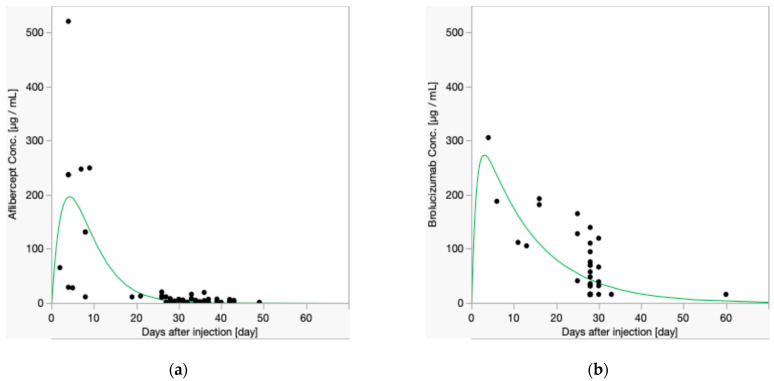
Concentrations of aflibercept and brolucizumab in the anterior chamber after each intravitreal injection. (**a**) Data for all aflibercept samples. The half-life of aflibercept was 2.88 days. (**b**) Data for all brolucizumab samples. The half-life of brolucizumab was 9.00 days. Samples were predominantly collected at 4 weeks post-injection, with fewer samples collected earlier and later, as shown in the figure.

The respective half-life graphs are presented in Figure 1. The overall half-lives were 2.88 (95% confidence interval [CI], from 2.02 to 5.06) days for aflibercept and 9.00 (95% CI, from 7.51 to 11.2) days for brolucizumab, demonstrating a statistically significant difference (*p* = 1.16 × 10^−7^).

### 2.3. Summary of Measurements Categorized by Dosage Interval

**Figure 2:**
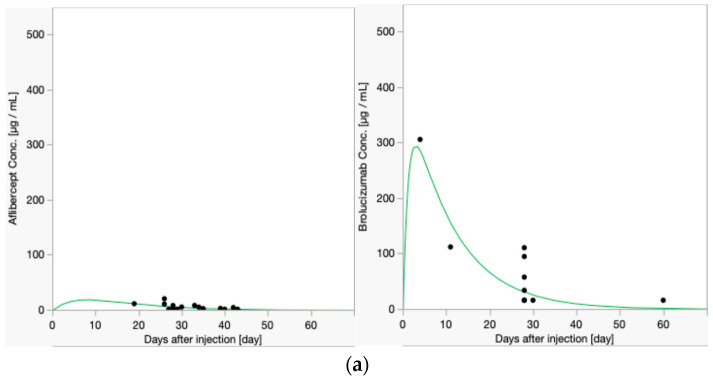

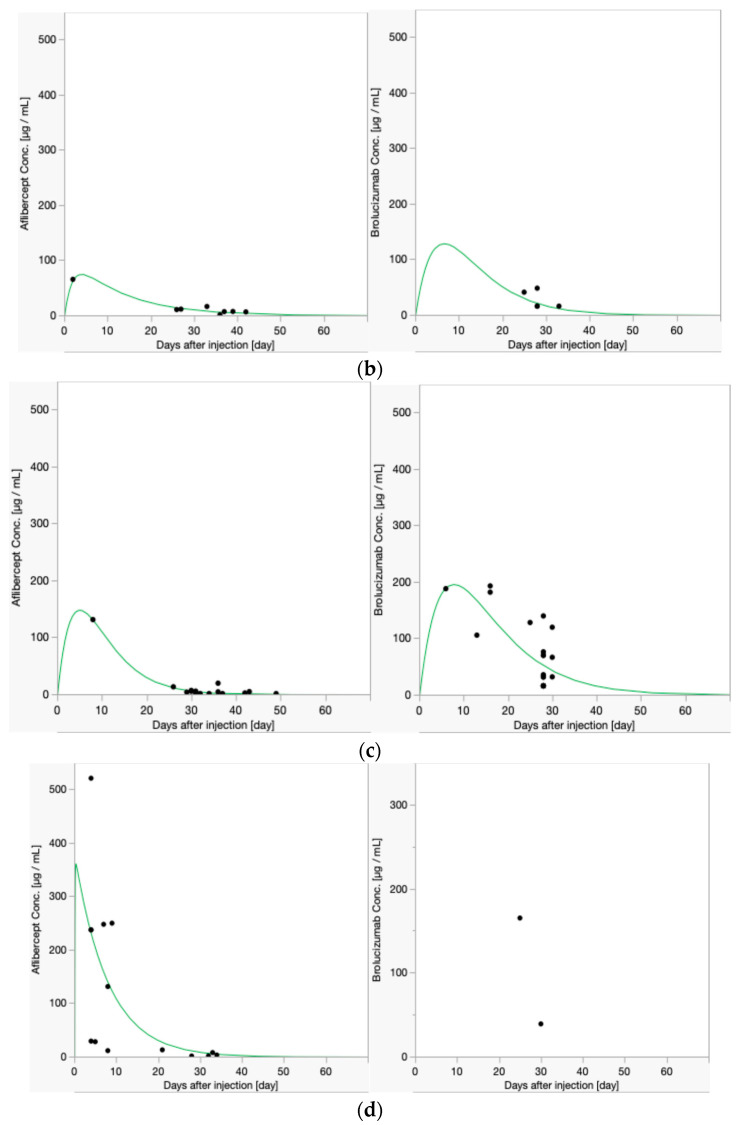
Concentrations of aflibercept and brolucizumab in the anterior chamber after each intravitreal injection. (**a**) Data for the “≤8-week interval group.” The half-life of aflibercept was 5.65 days and that of brolucizumab was 7.43 days. (**b**) Data for the “8-week<, and ≤ 16-week interval groups”. The half-life of aflibercept was 10.46 days and that of brolucizumab was 3.24 days. (**c**) Data for the “≥16-week interval group”. The half-life of aflibercept was 4.91 days whereas that of brolucizumab was 5.36 days. (**d**) Data for the “unknown treatment interval group”. The half-life of aflibercept was 5.51 days.

Patients were categorized as requiring frequent injections with intervals of less than 8 weeks and those requiring less frequent injections. The aqueous half-lives of both drugs were estimated. The half-life graphs for each group are shown in Figure 2.

As a result, the half-life of aflibercept in the vitreous was calculated as 5.65 (95% CI, from 3.54 to 14.00) days in the “≤8-week interval group”, 10.46 (95% CI, from 8.59 to 13.35) in the “8-week<, and ≤16-week interval group“, 4.91 (95% CI, from 4.21 to 5.88) in the “≥16-week interval group”, and 5.51 (95% CI, from −6.25 to 1.91) in the “unknown treatment interval group”. The half-life of brolucizumab in the vitreous was calculated as 7.43 (95% CI, from 5.72 to 10.59) in the “≤8-week interval group”, 3.24 (95% CI, from 1.63 to 273.37) in the “8-week<, and ≤16-week interval group”, and 5.36 (95% CI, from 4.42 to 6.82) in the “≥16-week interval group”. The half-life of aflibercept did not differ significantly when stratified by dosing interval, but that of brolucizumab was significantly longer in the “≤8-week interval group” compared to the “≥16-week interval group” (*p* = 0.002). Background diseases by dosing interval are shown in Table 2.

### 2.4. Factors Associated with the Half-Lives of Aflibercept and Brolucizumab

A multivariable analysis showed that no association was found for age, gender, ocular axis length, or PVD for aflibercept concentration (Table 3). For brolucizumab concentration, there was a positive association with age and a negative association with axial length and PVD (*p* = 0.0016, 0.019, and 0.03, respectively). The variance in inflation factors were under 3 for each factor.

## 3. Discussion

In this study, we calculated the half-lives of brolucizumab and aflibercept from aqueous humor concentrations using mass spectrometry. We found that the intravitreal half-life of brolucizumab was significantly longer than that of aflibercept (9.00 days vs. 2.88 days, *p* = 1.1 × 10^−7^). Additionally, the half-life of brolucizumab was significantly longer in the “≤8-week interval group” than in the “≥16-week interval group” (*p* = 0.002).

### 3.1. Development of LC-MS/MS Against ELISA and Its Significance

Numerous pharmacokinetic investigations have been conducted on aflibercept, a contrast to the limited number of studies on brolucizumab. Although there are many reports on intraocular pharmacokinetics of aflibercept, experimental conditions differ slightly, such as the test species used, i.e., rabbits or humans, and measurement methods, i.e., Biacore and ELISA [21,22,23,24]. Many studies on the pharmacokinetics of intravitreal injection drugs are conducted using ELISA. We previously reported that intraocular VEGF concentrations are underestimated in the presence of anti-VEGF reagents when measured using ELISA [19]. To circumnavigate this problem, we proposed a method of testing using mass spectrometry [20,21]. Similarly, Iwamoto et al. reported that the quantification of mAbs using ELISA is strongly influenced by endogenous ligands or anti-drug antibodies [25]. Mass spectrometry is not affected by anti-VEGF compounds and may more accurately measure VEGF concentrations.

### 3.2. Consistency of Brolucizumab and Aflibercept Measurements

**Figure 3:**
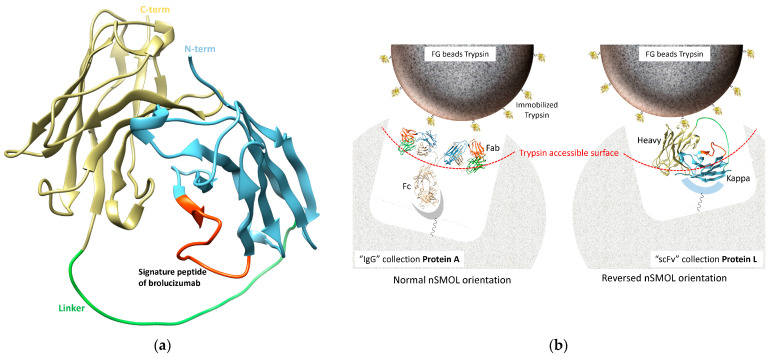
Reversed orientation of nSMOL reaction for the brolucizumab assay using Protein L collection resin. (**a**) Prediction of brolucizumab’s 3D structure by AlphaFold2 [26]. Three-dimensional structure prediction was performed on an AlphaFold2_mmseqs2 via Google Colab using 252 amino acids of brolucizumab. The 3D protein view was created with UCSF Chimera version 1.17.1 [27] using the rank 1 data of 3D prediction. The antigen-binding domain (complementarity-determining region) is arranged in the same conformation as the full-length IgGs. The N-terminal region of brolucizumab is shown in blue, and the C-terminal region is shown in yellow. The linker between the N- and C-termini is indicated in green. The signature peptide position of the brolucizumab assay is shown in red. (**b**) Schematic diagram of nSMOL proteolysis set in “normal” (left) and “reversed” (right) formats. Full-length IgGs and monoclonal antibodies are collected with Protein A resin via the Fc domain (“normal” nSMOL format), and the Fab is oriented to the tryptic reaction surface on FG bead nanoparticles. Brolucizumab is a short-chain, Fv-fragment antibody (scFv). scFvs are collected with Protein L via the kappa light chain (“reversed” nSMOL format), and the C-terminus of brolucizumab is oriented toward the reaction surface.

Brolucizumab is a Fab fragment with no Fc domain. Accordingly, we modified the assay (Figure 3). Specifically, we designed a reversed format nSMOL assay in which samples are collected on Protein L resin via the kappa light chain, and the C-terminus of brolucizumab is oriented toward the reaction surface, thus eliminating the need to use an Fc domain. Using this reversed format, the nSMOL method can generally be applied to all scFv antibodies that have no Fc domain. Consequently, we showed that mass spectrometry under the same conditions was able to measure the concentration of brolucizumab, enabling direct comparisons of the concentration and half-life of aflibercept with those of brolucizumab in patients who received anti-VEGF therapy.

### 3.3. Intravitreal Half-Lives of Aflibercept and Brolucizumab

The pharmacokinetics of aflibercept has been investigated using various approaches, including in vivo experiments with rabbits, after either intravenous or intravitreous injections. The results have been somewhat inconsistent. In plasma, the estimated half-life of free aflibercept was 5 to 6 days after intravenous administration, with doses ranging from 2 to 4 mg/kg [22], whereas in serum, the mean half-life for aflibercept following intravitreal administration was 11.4 ± 4.8 days [23]. In another study using the ELISA method on rabbits, the vitreous half-lives of aflibercept were 3.63 days [24], 4.33 days [28], and 4.71 days [29]. In contrast, there are still few reports on brolucizumab. Do et al. argued that the intraocular half-life of aflibercept obtained using human aqueous humor and also by ELISA was 11 days, but the sample size was five eyes [30]. The half-life of aflibercept in this study, 2.88 days, is slightly different from that in previous reports, but we believe it is within the acceptable range due to the different measurement methods used. In this study, 2 mg of aflibercept was employed. However, if 8 mg was used, the drug dosage would be four times higher. In such instances, the duration over which the effective concentration can be maintained would be twice the half-life. Consequently, based on the findings of this study, 8 mg doses of aflibercept might extend the dosing interval by approximately 6 days compared to the 2 mg dosage. Brolucizumab had a half-life of 9.00 days, which is consistent with its longer efficacy. However, considering its smaller molecular weight, these results were unexpected; however, they can be reconciled as described below.

Brolucizumab aggregates [31], whereas aflibercept does not [32]. These structural differences may affect the ease of physical elimination, which in turn may affect the half-life. In other words, the larger structure of brolucizumab, which forms aggregates, may make it more difficult to eliminate from the eye, resulting in a longer half-life. On the other hand, aflibercept has an Fc region in its molecular structure that is not present in brolucizumab; the binding of the Fc region to the FcRn receptor facilitates its transfer from the eye to systemic circulation [33]. These structural differences may affect the half-life.

In drug simulations, the concentrations of brolucizumab fall below the effective concentration faster than those of aflibercept [34], but in actual clinical practice, there are many reports that brolucizumab works longer and can extend the dosing interval [14]. One reason for this may be that brolucizumab forms a complex in the eye that slows down its clearance.

### 3.4. Results Classified by Brolucizumab Dosing Interval

Significant differences in half-life were observed between brolucizumab dosing intervals; the half-life was longer in the group with shorter dosing intervals and shorter in the group with longer dosing intervals. Normally, the shorter the half-life of a drug, the earlier the drug wears off; thus, the more frequently the drug must be administered. In other words, the half-life of the drug in the group requiring frequent dosing may be shorter, but the results of this study were the opposite. Although it is challenging to explain these results, it is possible that patients requiring frequent injections experience diminished drug efficacy, known as tachyphylaxis, which may alter intraocular pharmacokinetics. Tachyphylaxis is a phenomenon in which the effects of a drug diminish after repeated administration. This effect has been reported with anti-VEGF drugs such as bevacizumab, ranibizumab, and aflibercept [35,36,37,38,39]. The generation of neutralizing antibodies, or anti-drug antibodies, against those drugs has been identified as the cause of this phenomenon. Patients who require frequent dosing may be affected not only by the half-life of the drug but also by other factors such as tachyphylaxis.

### 3.5. Discussion of the Results from the Multivariate Analysis

A positive correlation with age and a negative correlation with ocular axis length and PVD were observed for brolucizumab. PVD is an age-related change in which the vitreous cortex separates from the inner retinal limiting membrane and is associated with the development of retinal diseases such as retinal detachment. The prevalence is 24% in patients aged 50–59 years but increases to 87% in patients aged 80–89 years [40]. In the elderly, a decrease in aqueous humor turnover rate is known to occur; thus, it is reasonable to observe a positive correlation with concentration. Moreover, since concentration decreases with increasing volume at the same component quantity, it is logical to observe a negative correlation between ocular axial length and concentration.

A previous study showed that human aqueous VEGF is lower in eyes with PVD than in those without [41]. Because the aqueous humor levels of cytokines correlate with vitreous levels [42], it is reasonable to speculate that the VEGF concentration in the vitreous is higher in eyes without PVD than in eyes with it.

### 3.6. Limitations

In this study, we used samples from all treated diseases to avoid an insufficient number of cases, which would have hindered the generation of a meaningful concentration curve. As a result, we calculated an average half-life across various diseases, although the half-life may vary depending on the specific condition.

All the samples in this study were obtained from Japanese patients, and this study was also biased toward elderly patients, such as those with nAMD. AMD, the most common underlying disease in this study, exhibits racial differences [43]. Therefore, the findings of this study may not be generalizable to all racial populations. Additionally, as the majority of patients in this study had AMD, the sample population predominantly consisted of elderly patients. Drug metabolism can vary with age, and younger patients may metabolize drugs more rapidly. Moreover, there were slight differences in underlying conditions between the aflibercept group and the brolucizumab group, which may have influenced intraocular pharmacokinetics.

Aflibercept is applicable to many diseases, and there were short-term data on NVG which were not available for brolucizumab. It has been reported that the concentration of intraocular VEGF in NVG is extremely high [44,45], and its binding to anti-VEGF antibodies may suppress the leakage of anti-VEGF antibodies. This may raise the initial anti-VEGF drug concentration in the graph with decreasing concentration, resulting in a shorter half-life of aflibercept. We did not exclude these cases due to the small sample size. The analysis of groups divided by the dosing interval resulted in an even smaller sample size and a larger disease bias.

Most samples were obtained during the second intravitreal injection, 4 weeks after the first injection. Samples collected beyond this period were often below the detection limit due to the low sensitivity of mass spectrometry, and the number of samples was small. Additionally, samples from earlier periods (before 4 weeks) rely heavily on patients who underwent cataract surgery, vitrectomy, or intravitreal injection, contributing to a smaller sample size in those cases as well. Due to the limited availability of early-phase data in the half-life curve, the half-life estimation may be significantly influenced by these few initial data points. Specifically, if the initial concentrations are high, the calculated half-life will be shorter, whereas lower initial concentrations will result in a longer half-life. The limited number of early phase data was insufficient to accurately calculate the half-lives.

In the future, by applying this method to populations with other diseases, establishing mass spectrometry techniques for other anti-VEGF agents, and comparing them with the ELISA method, it may be possible to further demonstrate the utility of mass spectrometry. This approach could provide a more thorough evaluation of pharmacokinetics, potentially enabling a better assessment of drug efficacy.

## 4. Materials and Methods

### 4.1. Reagents

nSMOL antibody BA kits (Shimadzu, Kyoto, Japan) were used for brolucizumab analysis. Modified trypsin-immobilized glycidyl methacrylate (GMA)-coated nanoferrite particle FG beads with surface activation by N-hydroxysuccinimide were purchased from Tamagawa Seiki Co., Ltd., (Nagano, Japan). The Toyopearl AF-rProtein L HC-650F resin was purchased from Tosoh Bioscience (Tokyo, Japan). The anti-VEGF molecule, brolucizumab, was purchased from Novartis Biopharma (Basel, Switzerland). Control human serum was obtained from Kohjin Bio (Saitama, Japan). The synthetic peptide, P14R (14 Pro and Arg), octyl-β-D-glucopyranoside (OTG), LC-MS-grade organic solvents, and other reagents were obtained from Sigma-Aldrich (St. Louis, MO, USA) and Fuji Film Wako Chemicals (Osaka, Japan).

### 4.2. Structure Identification of Signature Peptides for Brolucizumab

Digestion Procedure

1. Preparation: a total of 2 µg of brolucizumab was diluted in 100 µL of nSMOL-enhanced solution;
2. Digestion: the solution was digested using FG beads Trypsin-DART (Shimadzu) at 50 °C for 5 h, with gentle vortexing in a saturated vapor atmosphere;
3. Termination: digestion was halted by adding 10% trifluoroacetic acid (TFA) to a final concentration of 0.5%.

Peptide Cleanup

1. Desalting: tryptic peptides were desalted on a MonoSpin C18 column (GL Science, Tokyo, Japan).
  ○ Column equilibration was performed using 0.1% TFA at 700× *g* for 30 s;
  ○ Peptides were then eluted with 70% acetonitrile containing 0.1% TFA and completely dried using a centrifugal evaporator.

LC-MS/MS Analysis for Structure Identification

1. Sample Preparation: dried peptides were reconstituted in 0.1% aqueous formic acid (FA);
2. Injection: a total of 1 µg of brolucizumab digest was injected into an LC-MS/MS system with a high-resolution electrospray ionization (ESI) quadrupole time-of-flight (Q-TOF) mass spectrometer (LC-Mikros and LCMS-9030, Shimadzu).

LC Conditions

- Solvent A: 0.1% aqueous FA;
- Solvent B: 0.1% FA in Acetonitrile;
- Trap Column: YMC-Pack Pro (YMC, Kyoto, Japan), 0.3 × 5 mm, 5 µm particle diameter;
- Separation Column: L-column2 (CERI, Tokyo, Japan), 0.3 × 150 mm, 2 µm particle diameter;
- Column Temperature: 40 °C;
- Flow Rates: 40 µL/min (trap column), 3 µL/min (separation column);
- Gradient Program:
  ○ 0–3 min: %B = 1;
  ○ 3–5 min: %B = 3 to 10 (linear gradient);
  ○ 5–50 min: %B = 10 to 40;
  ○ 50–55 min: %B = 100;
  ○ 55–65 min: %B = 1.

MS/MS Conditions

1. Data Acquisition: MS data were collected from 8 to 50 min, covering an *m/z* range of 350 to 1000 for MS and 50 to 1500 for MS/MS;
2. Ionization Parameters:
  ○ Interface: Electrospray, 100 °C;
  ○ DE solvation Line: 250 °C;
  ○ Heat Block: 400 °C;
  ○ Nebulizer Gas Flow: 2 L/min;
  ○ Heating Gas Flow: 3 L/min;
  ○ Collision Cell: 250 kPa of argon at 25 V ± 5 V.

Peptide Identification

1. Database Search: peptide identification was performed using Peaks Studio Xpro, version 10.5, with the following criteria:
  ○ Mass Tolerances: A total of 25 ppm for parent mass, 50 mDa for fragment mass;
  ○ False Discovery Rate: 5%;
  ○ Modifications: oxidized methionine, pyroglutamate on the N-terminus.

Signature Peptides Selection for Quantification

1. Identified Peptides:
  ○ Peptide 1: LTVLGGGGGS GGGGSGGGGS GGGGSEVQLV ESGGGLVQPG GSLR (aa.108–151), *m/z* 907.204, [M+4H]4+ (Supplementary Figure S1);
  ○ Peptide 2: LLIYLASTLA SGVPSR (aa.47–62), *m/z* 830.99, [M+2H]^2+^ (Supplementary Figure S1).

### 4.3. Sequence Alignment of Brolucizumab Signature Peptides

The sequences of the signature peptides for brolucizumab were aligned with ClustalW using the sequences of brolucizumab (KEGG Drug entry no. D11083, https://www.kegg.jp/entry/D11083 accessed on 23 April 2021.), bevacizumab (DrugBank no. DB00112, https://go.drugbank.com/drugs/DB00112 accessed on 17 December 2014), and trastuzumab (KEGG Drug entry no. D03257) on GENETYX software version 13.1.2 (GENETYX, Tokyo, Japan). The peptide LTVLGGGGGS GGGGSGGGGS GGGGSEVQLV ESGGGLVQPG GSLR was assigned as a part of the linker region between the heavy and light chains of brolucizumab, and the peptide LLIYLASTLA SGVPSR was identified as a candidate-specific sequence (Supplementary Figure S2).

### 4.4. Optimization of Multiple Reaction Monitoring (MRM) Quantification for Brolucizumab Signature Peptides

Tryptic peptides of brolucizumab were prepared as described above. Two candidate signature peptides were analyzed using ESI triple quadrupole (TQ) mass spectrometry coupled with high-performance liquid chromatography (Nexera ×2 and LCMS-8060, Shimadzu). Method optimization for peptide quantification for the pre-bias electrode of quadrupoles 1 and 3 from −50 to −25 V, the collision cell electrode from −50 to −10 V, and the *m/z* of fragment ions were performed using the automated software tool in LabSolutions (Shimadzu). LC-MS conditions were as follows: solvent A, 0.1% aqueous FA; solvent B, 0.1% FA in acetonitrile; column, Shim-pack (Shimadzu) GISS C_18_, 2.1 × 50 mm, 20 nm pore size, and 1.9 µm particle diameter; column temperature, 50 °C; and flow rate, 0.4 mL/min. The gradient program was as follows: 0 to 1.5 min, %B = 1; 1.5 to 2.5 min, %B = 1 to 28 with a linear gradient; 2.5 to 5.0 min, %B = 28 to 33 gradient; 5.0 to 6.0 min, %B = 95; and 6.0 to 7.0 min, %B = 1. The temperatures of the ESI probe, de-solvation line, and heat block were 300 °C, 250 °C, and 400 °C, respectively. Nebulizer, heating, and drying gas flows were set to 3, 10 and 10 L/min, respectively. The electrode of the ESI interface was set to +4.0 kV. Argon gas pressure in the collision cell for MS/MS was set to 270 kPa. The peptide LLIYLASTLA SGVPSR was finally identified as a signature peptide for brolucizumab quantitation. The MRM transition of *m/z* 831.0 for parent [M+2H]^2+^ and *m/z* 359.10 (y3^+^) for the fragment ion was set as a quantitation channel, and *m/z* 831.0 for parent and *m/z* 602.4 (y6^+^) and 673.4 (y7^+^) fragments were set as structure confirmation channels (Supplementary Figure S3).

### 4.5. Participants

At the outpatient clinic of the Ophthalmology Department at Jichi Medical University and JCHO Tokyo Shinjuku Medical Center, we collected aqueous humor from consenting patients at the time of injection and at the time of surgery as part of a prospective biomarker study [37]. In this study, we used 111 stored aqueous humor samples collected from March 2013 to August 2021. Patient disease background information included proliferative diabetic retinopathy (PDR), NVG, branch retinal vein occlusion (BRVO), and nAMD). There were 59 samples collected after aflibercept injection, including 48 nAMD, 5 NVG, 5 PDR, and 1 BRVO, as well as 52 samples collected after brolucizumab injection, all with nAMD. Axial length was measured with an OA-2000 (TOMEY, Nagoya, Japan). The presence or absence of PVD was evaluated using DRI OCT Triton plus, (Topcon, Tokyo, Japan) by retinal specialists (KN and HT).

### 4.6. Quantification of Aflibercept in Human Aqueous Humor

All samples of human aqueous humor were prepared and stored at −80 °C for at least 24 h before nSMOL assay. The concentrations of aflibercept were measured using our previously reported method [20].

### 4.7. Quantification of Brolucizumab in Human Aqueous Humor

In our earlier study, we performed the antibody analysis using the nSMOL (nano-surface and molecular-orientation limited proteolysis) platform. For selective detection of specific peptide sequences in complementarity-determining regions (CDRs) in the Fab chains of monoclonal antibodies, nSMOL enables limited access of protease to antibody substrates and selective proteolysis of the Fab by collecting the antibody via Fc in 100 nm pores of Protein A resin to orient the Fab toward the reaction solution and immobilizing the protease on the surface of nanoparticles with a 200 nm diameter. However, brolucizumab is a Fab fragment with no Fc domain, so the assay was performed using our previously reported methods with minor modifications (Figure 3). The Brolucizumab concentration in human aqueous humor was measured by diluting it 1:4 in pooled human plasma. The antibody fraction from human aqueous humor diluted with plasma was collected via the ĸ-light chain using 12.5 μL of PBS-substituted Protein L resin slurry (25%) in 100 μL of PBS containing 0.1% OTG with gentle vortexing at 25 °C for 5 min on an Ultrafree PTFE 0.2 µm filter device (Millipore, Burlington, MA, USA). The Protein L collection resin was washed twice with 200 μL of PBS containing 0.1% OTG to remove the other diluted aqueous humor proteins except for immunoglobulins and then washed twice more with 200 μL of PBS to remove detergents that can inhibit column separation and the ionization of peptides. After this washing, the Protein L resin was suspended in 80 μL of the nSMOL-enhanced reaction solution containing 5 fmol/µL of P14R internal standard, and the suspension was immediately transferred into a Protein LoBind tube (Eppendorf, Hamburg, Germany). The nSMOL reaction was carried out using 5 μg of trypsin on FG beads with gentle vortexing at 50 °C for 5 h in a saturated vapor atmosphere for uniform contact between the collection resin and FG bead nanoparticles. After nSMOL proteolysis, the reaction was quenched by adding 10% FA at a final concentration of 0.5%. The peptide solution was collected by centrifugation (10,000× *g* for 1 min) on an Ultrafree 0.2 µm filter to remove the collection resin and the trypsin FG beads. The filtered analyte solution was further purified via magnetic separation from the residual FG beads, and the supernatant was transferred to a TORAST-H bio vial (Shimadzu GLC, Tokyo, Japan) for MS analysis. The lower limit of quantitation of brolucizumab in human diluted aqueous humor was 0.391 µg/mL.

### 4.8. Patient Classification of Pharmacokinetic Half-Lives

Almost all nAMD patients had been treated with a treat-and-extend regimen. Some were treated on an as-needed basis. Patients in which the dosing interval can be extended indicate a positive therapeutic response, whereas patients requiring shorter intervals suggest a more refractory response to the treatment. We conducted a subgroup analysis by categorizing patients into four categories based on treatment intervals to investigate whether these differences reflect variations in intraocular pharmacokinetics or other variables, such as the severeness of the diseases.

The first cohort comprised patients who were unable to prolong the treatment interval beyond 8 weeks, designated as the “≤8-week interval group”. The second cohort consisted of those who successfully extended the treatment interval to at least 16 weeks, termed the “≥16-week interval group”. The third cohort consisted of those with treatment intervals between 8 and 16 weeks, “8-week<, and ≤16-week interval group“. The fourth cohort consisted of those whose treatment interval was unknown, “unknown treatment interval group”. The eyes underwent a change in treatment before the establishment of a fixed interval as per the treat-and-extend regimen.

### 4.9. Statistical Analysis

The pharmacokinetic half-lives of the agents under investigation were computed employing a one-compartment model, using JMP Pro 17.2.0 statistical software (SAS Institute Inc., Cary, NC, USA). Subsequently, intergroup variances in half-lives were evaluated using Student’s *t*-test to determine the significance of observed differences. A multivariate analysis was conducted on both the aflibercept and brolucizumab groups, employing multiple regression analysis to examine concentrations in relation to variables such as gender, age, the axial length of the eye, and the presence of PVD. Samples below the limit of quantitation were calculated using half of the minimum quantitation limit value.

## 5. Conclusions

Using the same mass spectrometry conditions, the half-lives of aflibercept and brolucizumab in the anterior chamber of the eye were calculated as 2.88 and 9.00 days, respectively, and we believe that this ratio is useful for discussion on the efficacy and duration of both drugs. We believe that the validity of mass spectrometry will be demonstrated through further studies, including comparisons with ELISA, establishing mass spectrometry with other anti-VEGF drugs, and measuring pharmacokinetics in other diseases to demonstrate the accuracy of pharmacokinetics using mass spectrometry. In addition, we believe that pharmacokinetic studies using mass spectrometry will advance discussions of drug efficacy and duration.

## Figures and Tables

**Table 1:** Demographic characteristics of patients who received aflibercept or brolucizumab.

	Aflibercept (59)	Brolucizumab (52)	*p* Value
Age, (year, SD)	70.8 (10.3)	74.7 (7.4)	0.03 *
Sex, male (%)	45 (76)	38 (73)	0.70
Axial length (mm, SD)	23.8 (0.98)	23.5 (0.76)	0.23
Posterior vitreous detachment (+, %)	21 (36)	32 (62)	0.006 *

*: *p* < 0.05.

**Table 2:** Background diseases of patients who received aflibercept or brolucizumab by administration interval.

Interval of Injections	Disease	Aflibercept (59)	Brolucizumab (52)
All	AMD	48	52
	NVG	5	
	PDR	5	
	BRVO	1	
<8week	AMD	20	15
8-week<, and ≤16 weeks	AMD	7	11
	BRVO	1	
≤16 weeks	AMD	16	24
	NVG	1	
unknown	AMD	5	2
	NVG	4	
	PDR	5	

AMD: age-related macular degeneration, NVG: neovascular glaucoma, PDR: proliferative diabetic retinopathy, BRVO: branch retinal vein occlusion.

**Table 3:** Partial regression coefficients between demographic characteristics and half-lives, using a multiple regression analysis.

	Aflibercept		Brolucizumab	
	T-Value	*p* Value	T-Value	*p* Value
Age (year)	−1.8	0.17	4.0	0.0016 *
Sex (male)	16.1	0.27	−5.6	0.51
Axial length (mm)	−4.0	0.80	−35.1	0.019 *
Posterior vitreous detachment (+)	13.4	0.34	−20.1	0.030 *

*: *p* < 0.05.

## Data Availability

The original contributions presented in this study are included in the article/Supplementary Materials; further inquiries can be directed to the corresponding author.

## Supplementary Materials

The following supporting information can be downloaded at: https://www.mdpi.com/article/10.3390/ijms26020556/s1.
