# Supplementary material for: Comparative Pharmacokinetic Analysis of Aflibercept and Brolucizumab in Human Aqueous Humor Using Nano-Surface and Molecular-Orientation Limited Proteolysis
Source: Int J Mol Sci. 2025 Jan 10;26(2):556. doi: 10.3390/ijms26020556 (PMC11764841; doi:10.3390/ijms26020556)
Supplement: Supplementary file 1 [file ijms-26-00556-s001.zip › ijms-3273662-supplementary.pdf]

Supplementary information

Comparative Pharmacokinetic Analysis of Aflibercept and Brolucizumab in Human Aqueous Humor Using Nano-Surface and Molecular-Orientation Limited Proteolysis

Kosuke Nagaoka [1,†], Natsuka Kimura [2,†], Satoru Inoda [1], Takuya Takayama [1], Yusuke Arai [1], Yasuo Yanagi [3,4],
Takashi Shimada [5], Ryozo Nagai [6], Hidenori Takahashi [1] and Kenichi Aizawa [2,7,8,*]

[1] Department of Ophthalmology, Jichi Medical University, Shimotsuke-shi 329-0498, Tochigi, Japan; knagaoka@jichi.ac.jp (K.N.); r1208is@jichi.ac.jp (S.I.); takayama.takuya@jichi.ac.jp (T.T.); r1003ya@jichi.ac.jp (Y.A.); takahah@jichi.ac.jp (H.T.)
[2] Division of Clinical Pharmacology, Department of Pharmacology, Jichi Medical University, Shimotsuke-shi 329-0498, Tochigi, Japan; kimura_n@jichi.ac.jp
[3] Department of Ophthalmology and Micro-Technology, Yokohama City University, Yokohama-shi 232-0023, Kanagawa, Japan; yanagi.yas.wu@yokohama-cu.ac.jp
[4] Singapore National Eye Centre, Singapore Eye Research Institute, Singapore 168751, Singapore
[5] Technology Research Laboratory, Shimadzu Corporation, Kyoto-shi 604-8511, Kyoto, Japan; tshima@shimadzu.co.jp
[6] Jichi Medical University, Shimotsuke-shi 329-0498, Tochigi, Japan; rnagai@jichi.ac.jp
[7] Clinical Pharmacology Center, Jichi Medical University Hospital, Shimotsuke-shi 329-0498, Tochigi, Japan
[8] Division of Translational Research, Clinical Research Center, Jichi Medical University Hospital, Shimotsuke-shi 329-0498, Tochigi, Japan
* Correspondence: aizawa@jichi.ac.jp; Tel.: +81-285-58-7388; Fax: +81-285-44-7562
† These authors contributed equally to this work.

a)

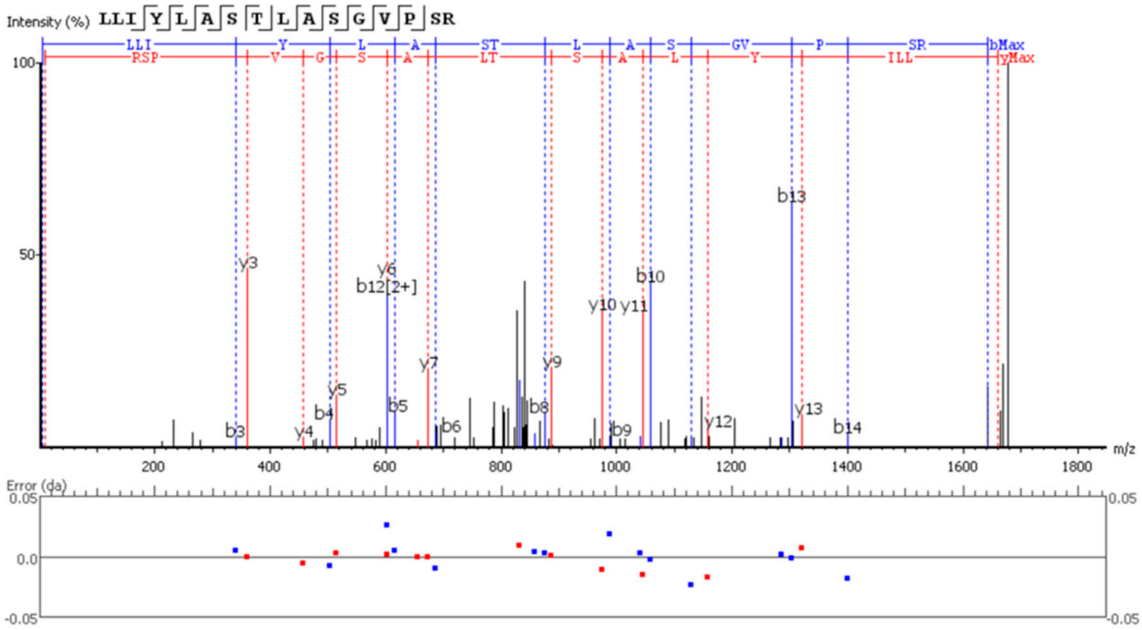

b)

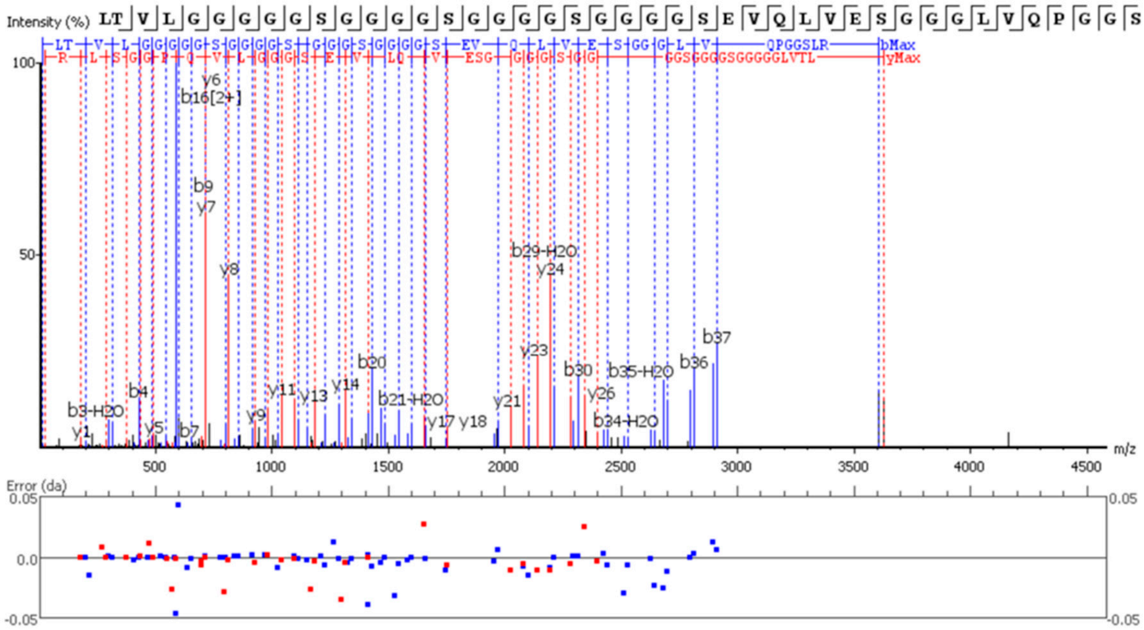

Supplementary Figure S1. (a) Sequence assignment of LLIYLASTLA SGVPSR (aa.47-62, m/z 830.99, [M+2H]2+) by MS/MS database search. The upper figure shows the assignment of amino acid sequences on MS/MS peak profile, and the lower box shows the error plot [Da] of each MS/MS ion for b- (blue) and y-ion (red) series. (b) Sequence assignment of LTVLGGGGGS GGGGSGGGGS GGGGSEVQLV ESGGGLVQPG GSLR (aa.108-151, m/z 907.204, [M+4H]4+) by MS/MS database search.

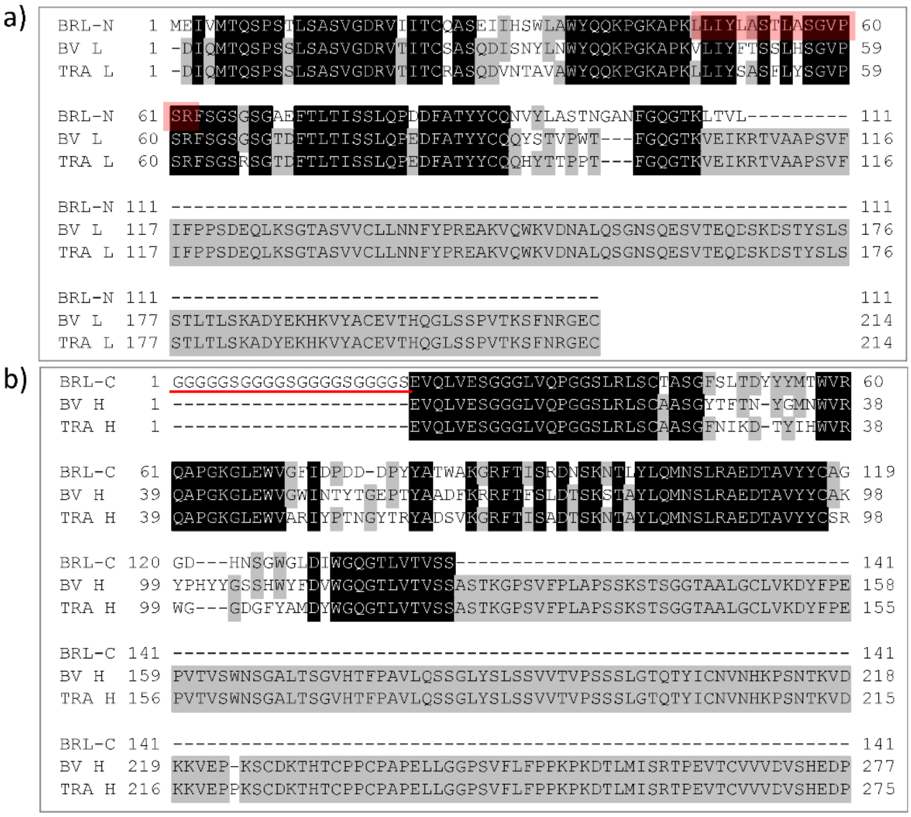

Supplementary Figure S2. Multiple sequence alignment of brolucizumab a) N-terminal (BRL-N), and b) C-terminal (BRL-C) domain with trastuzumab (TRA Land H chain) and bevacizumab (BV Land H chain). The red highlight shows the candidate signature peptide, and the underline show the inserted linker sequence between BRL-N and BRL-C.

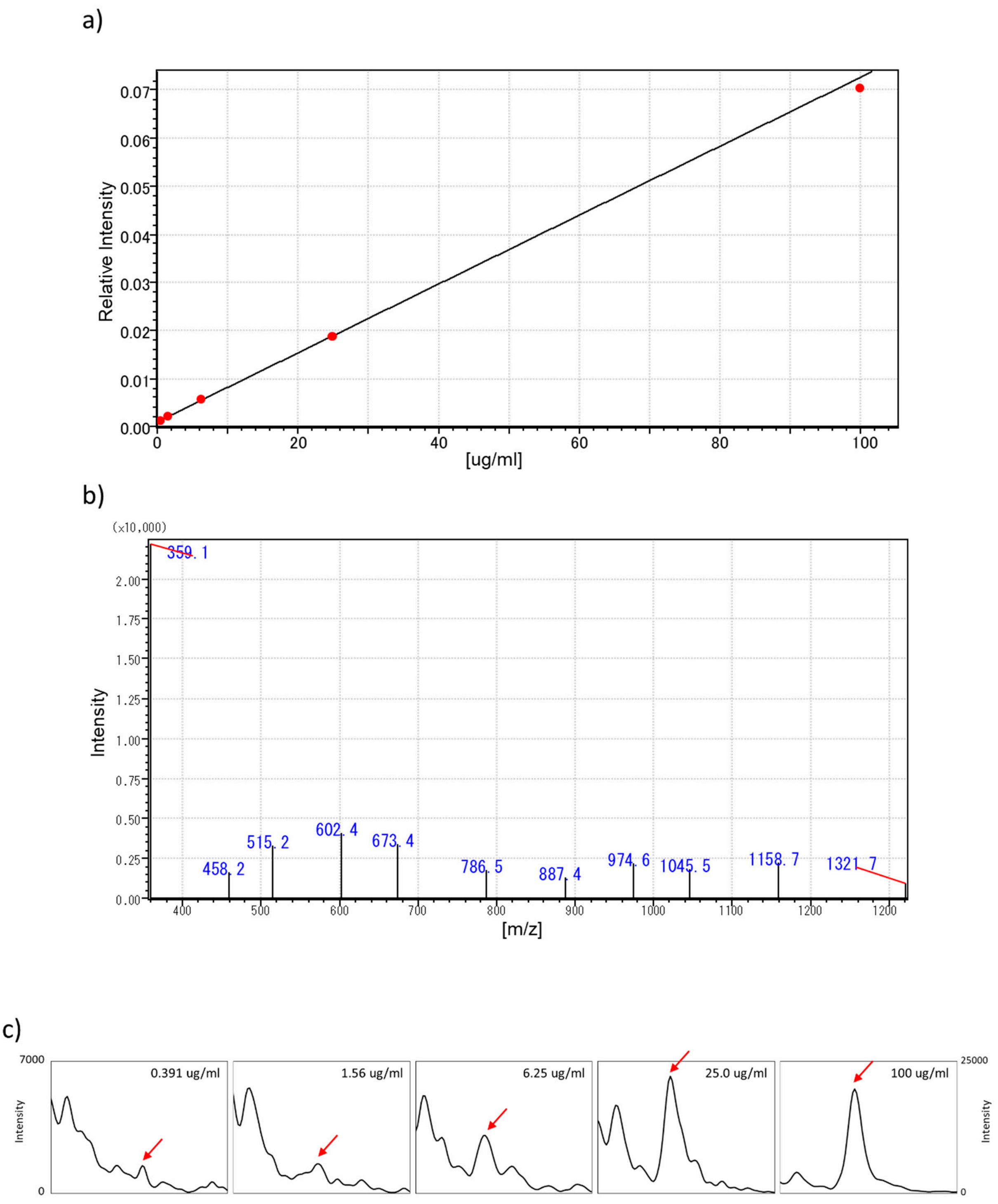

Supplementary Figure S3. (a) A representative calibration standard of LLIYLASTLA SGVPSR (parent m/z 831.0 and fragment m/z 359.1 [y3+]) from 0.391 to 100 µg/mL of brolucizumab in human serum by nSMOL assay coupled with LCMS MRM analysis. The coefficient of determination (R^2) was 0.973 for concentrations between 0.391 and 100 µg/mL, corresponding to brolucizumab in human serum. (b) Representative MRM spectra of each transition from LLIYLASTLA SGVPSR (fragment m/z 359.1, 515.2, 602.4, 673.4, 786.5, 887.4, 974.6, 1045.5, and 1321.7). (c) Representative extracted ion

chromatogram (XIC) for MRM channel (m/z 831.0>359.1) on calibration brolucizumab samples of 0.391, 1.56, 6.25, 25, and 100 μg/ml in human serum. The red arrow on RT 3.65 min is indicated the MRM signal from LLIYLASTLASGVPSR. Y axis on left is shown for samples of 0.391, 1.56, 6.25, 25 μg/ml, and on right for 100 μg/ml.
